# Information and communication technology to enhance continuing professional development (CPD) and continuing medical education (CME) for Rwanda: a scoping review of reviews

**DOI:** 10.1186/s12909-021-02607-w

**Published:** 2021-04-29

**Authors:** Joseph Lune Ngenzi, Richard E. Scott, Maurice Mars

**Affiliations:** 1 Department of Telehealth, School of Nursing & Public Health, College of Health Sciences, University of KwaZulu-Natal, Durban, South Africa; 2 Department of Health Informatics, University of Rwanda, Kigali, Rwanda; 3 Department of Community Health Sciences, Cumming School of Medicine, University of Calgary, Calgary, Alberta Canada; 4 College of Nursing and Health Sciences, Flinders University, Adelaide, South Australia Australia

**Keywords:** Information and communications technology (ICT), Technology enabled and enhanced teaching (TEET), Continuing medical education (CME), Continuing professional development (CPD), lifelong learning, Rwanda, Developing Countries

## Abstract

**Background:**

Access to high quality continuing professional development (CPD) is necessary for healthcare professionals to retain competency within the ever-evolving worlds of medicine and health. Most low- and middle-income countries, including Rwanda, have a critical shortage of healthcare professionals and limited access to CPD opportunities. This study scoped the literature using review articles related to the use of information and communication technology (ICT) and video conferencing for the delivery of CPD to healthcare professionals. The goal was to inform decision-makers of relevant and suitable approaches for a low-income country such as Rwanda.

**Methods:**

PubMed and hand searching was used. Only review articles written in English, published between 2010 and 2019, and reporting the use of ICT for CPD were included.

**Results:**

Six review articles were included in this study. Various delivery modes (face to face, pure elearning and blended learning) and technology approaches (Internet-based and non-Internet based) were reported. All types of technology approach enhanced knowledge, skills and attitudes. Pure elearning is comparable to face-to-face delivery and better than ‘no intervention’, and blended learning showed mixed results compared to traditional face-to-face learning. Participant satisfaction was attributed to ease of use, easy access and interactive content.

**Conclusion:**

The use of technology to enhance CPD delivery is acceptable with most technology approaches improving knowledge, skills and attitude. For the intervention to work effectively, CPD courses must be well designed: needs-based, based on sound educational theories, interactive, easy to access, and affordable. Participants must possess the required devices and technological literacy.

## Background

Effective healthcare service delivery requires qualified healthcare professionals (those who can provide healthcare, treatment, and advice based on formal training and experience) practising evidence-based medicine [1]. Health and healthcare delivery are not static, leading to shifts in the evidence-base of medicine. As a result changes to service delivery, patient management, and personal wellness management constantly take place. In 2011, Densen projected that by 2020 medical knowledge would double every 73 days [2] and given the average career is 30 years for medical doctors and 40 years for nurses [3], maintaining knowledge and skills is a constant challenge. The concept of life-long learning has arisen as a means of accommodating this constant change. Lifelong learning has been defined as “all learning activity undertaken throughout life, to improve knowledge, skills, and competences within a personal, civic, social and employment-related perspective” [4]. In many low- and middle-income countries, access to lifelong learning is a challenge, including for overburdened healthcare professionals in isolated rural or remote locations [5].

One approach to providing access and encouraging the practice of life-long learning is to ensure healthcare providers have the opportunity to participate in structured continuing professional development (CPD) or continuing medical education (CME) activities throughout their careers. Furthermore, the sudden and unexpected impetus to pursue virtual solutions as a consequence of COVID-19 means the application of information and communication technologies (ICT) to provide effective CME and CPD is of even more immediate concern.

Within the context of this paper CME is considered a component of CPD, with CME focusing on improving medical knowledge and skills of health professionals and CPD providing additional skills required to practise high-quality medicine, including teaching, leadership, ethical, social, and personal skills [6, 7]. This type of education can be mandatory or voluntary [8]. Still, the main goal is to ensure currency of ability that supports and maintains quality and competence of those who provide healthcare services and patient management [5, 8]. To facilitate further presentation in this paper, when CPD is referenced, it will be considered to encompass CME also.

Historically the common formats for delivery of CPD have been through local face-to-face ‘in-service’ workshops or presentations, or presentations at larger academic conferences. More recently, world-wide use of ICT became common [9], and different phases in technology use have been seen. Initially, educational material was made available on personal computers or laptops and was termed ‘computer-based training’ (CBT) or ‘computer-based learning’ (CBL). With the introduction of compact discs (CDs) and digital video discs (DVDs), CD/DVD-based training followed, providing reading material, modular programmes, and recorded lectures. With the advent of the publicly available Internet, and soon after the World Wide Web (www), ‘elearning’ (defined as the delivery of training material via ICT) became truly interactive, allowing one-to-many approaches such as videoconferencing, online modules, and Massive Open Online Courses (MOOCs). Since that time, innovations such as wireless and smartphone technologies have shifted elearning towards mobile learning which, again because of the COVID-19 pandemic, is rapidly growing in application [10].

Another term has been applied to reflect the different contributions of technology – Technology Enabled and Enhanced Training (TEET) [11]. Technology both *enables* training in terms of connectivity by various means (e.g., mobile learning), but also *enhances* the training through the use of more interactive tools (e.g., video; graphics; images; simulation). Delivery of such programmes is often facilitated by the use of Learning Management Systems (LMS) such as Moodle, Blackboard, Desire2Learn, Sakai, Docebo, and A Tutor [12]. These now provide interactive and automated access to many different types of learning content, such as reading materials, video and audio recordings, wikis, web conferencing, chats, forums, blogs, learning games, testing, and grading tools [13]. TEET continues to evolve, given its close alignment with growing technology options.

Several TEET solutions are being used in healthcare. Delivery can be asynchronous (store and forward; e.g., modules accessed online at convenient times) or synchronous (real-time; e.g., audio or videoconferencing). When face-to-face and some form of TEET are used to complement each other, it is termed ‘blended learning’. Alternatively, when no face-to-face component is included, it is termed ‘fully online’ or ‘purely online’ [14, 15]. When TEET-based assignments are performed before a face-to-face component, it is termed a ‘flipped classroom’ format [15].

Rwanda is a small landlocked country located in the Great Rift Valley in East and Central Africa, with a young, mainly rural, but highly-dense population of 12.3 million in just 26,338 km^2^. In the wake of the 1994 Tutsi genocide, the country recovered and strengthened economically, as well as in terms of its health status. Most millennium development goals (MDGs) have been achieved, and targets for the sustainable development goals (SDGs) are being pursued.

Like many low and middle-income countries, Rwanda suffers from critical shortages, inadequate skill mixes, and uneven geographical distribution of its existing health workforce. The World Health Organization has identified 23 doctors, nurses and midwives per 10,000 population as the minimum level of healthcare providers required to deliver essential maternal and child health services. Rwanda has 1.3 doctors, and 12 nurses and midwives per 10,000, roughly half the minimum number required [16]. Inadequate skill mix has been documented in Rwanda [17] but its extent and impact was well demonstrated in Nepal where appropriate skill mix was documented as “particularly very low in observed health facilities” and it was observed to have a ‘knock-on’ effect with “absence of one category of health workers negatively impact[ing] on the performance of other health workers which affects health delivery system as a whole” [18]. Uneven distribution of human resources for health is also apparent in Rwanda, where it is reported that 82% of the population resides rurally, but only 12% of physicians and 42% of nurses serve in rural areas [19].

Nationally many government services are being actively digitised, including those within the education and health sectors [20]. CPD is now mandatory for healthcare professionals (https://www.rahpc.org.rw/cpd). The government wishes to implement innovative ICT approaches to deliver CPD, and has recently invested in videoconferencing. Overall, the government of Rwanda sees the use of ICT as one key strategy by which to achieve all development needs of the country [21].

However, the breadth of technological options, andragogical principles, and educational formats, and their combinations and permutations, are vast. Understanding which alternatives to apply in any setting is complex and must align available options with local or country context and need. Given the mandatory requirement for CPD in Rwanda, options for use of ICT for CPD need to be investigated.

This study aimed to scope the literature to identify available and successful TEET options for delivery of CPD to healthcare professionals. The insight gained will inform policy and decision-makers to determine which TEET options might be most suitable for providing CPD in the context of a low-income country such as Rwanda, with limited and widely distributed healthcare professionals.

## Methods

To document and understand the scope of ICT use for CPD a scoping review was undertaken. The stages included: 1) identifying the research question, (2) identifying relevant studies, (3) study selection, (4) charting the data, and (5) collating, summarising, and reporting results. Only reviews were considered, and only the PubMed database was searched. The search was conducted on 31 December 2019. The following broad search string was used: (Education, Medical, Continuing OR Continuing Professional Development) AND (Information Technology OR Videoconferencing). Videoconferencing was specifically included as it is a focus of the Rwanda Government. Searches were filtered by ‘review’ article type and for the 10 years 2010 to 2019, inclusive. The PubMed searches were supplemented by hand searching of government documents from Rwanda and the references lists of the selected papers.

Duplicates were removed before titles and abstracts were reviewed by all authors using the inclusion criteria: English language; review; and addressed use of ICT for CPD (or CME) of healthcare professionals. Inclusion for full paper review was determined by consensus, and all authors reviewed the full-text papers.

The following data were extracted and charted from each paper: author, publication year, title, country of study, the aim of the study, health discipline(s), number of cited articles, learning theories, the technology used, delivery mode, and major finding(s). Categories and themes were determined based on the review’s major and secondary outcomes relevant to the study objectives.

## Results

**Fig. 1:**
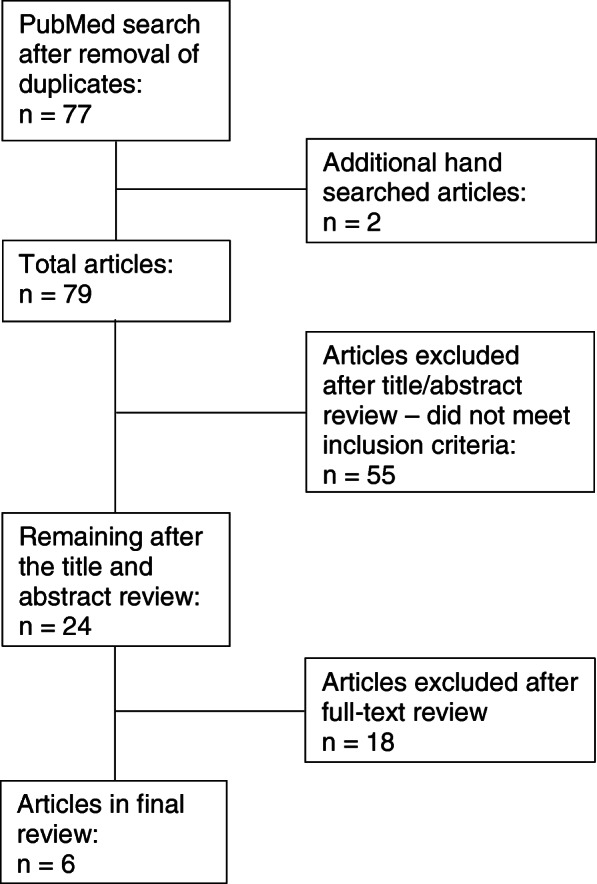
PRISMA flowchart of search results

Of the 79 unique papers, six were included in the study (Fig. 1). Four were systematic reviews [22–25] and two were integrated reviews [26, 27]. One paper addressed only nurse preceptors [25] and another only rural allied health personnel [22]. The remainder targeted multiple health disciplines, including nurses, allied health personnel, medical doctors, and pharmacists [24, 26–28].

Examining the reference lists of the included studies and discarding duplicates, the six articles reviewed 88 primary and unique studies. Thirty-one of these articles originated from North America (The United States of America (USA) - 24; Canada - 7), while 15 articles originated from Europe and 11 from Australia. Thus, more than half of the studies were from more developed countries than Rwanda. The remaining papers came from Asia and South America, with only two from Africa.

Three of the included reviews reported studies where a wide variety of learning theories had been used to design CPD [23, 25, 26]. These theories included Bandura’s theory of self-efficacy, Benner’s model from novice to expert, cognitive behavioural therapy, Rogers’ 5 stage innovation theory, the theory of planned behaviour, the extended theory of planned behaviour, interventional mapping, social learning theory, experiential learning, and problem-based learning theory.

Three themes (modes of delivery, enabling technology (facilitating delivery) and enhancing technology (facilitating presentation and content)) and categories of mode or technology were determined. A range of modes of delivery and technologies were used for providing CPD by TEET (Table 1).

**Table 1:** Summary and Categorisation of Reported Modes of Delivery and Technologies Used

	Description	Berndt et al. (2017)	Lawn et al. (2017)	Rohwer et al. (2017)	Sullivan (2017)	McLoughlin et al. (2018)	Wu et al. (2018)
**Mode of Delivery**	The way training instructions are delivered to support and enable the learning process.						
Face - to - face / On-campus delivery	A mode of study where lectures and other coursework is delivered physically in the traditional classroom/laboratory setting at scheduled times. Note: On-campus students will typically have access to the same online class-space as students studying online or via blended delivery.	X^*^		X	X		X
Blended delivery	A mode of study which encompasses both online and face-to-face learning.		X	X	X		X
Pure online delivery	A mode of study in which there are no face-to-face interactions and all learning materials are available online.		X	X	X	X	
**Technological approach**							
**1. Enabling Technology**							
*Not Internet-mediated* *-* CBT / CBL - CD, DVD, memory stick	Engaging participants at different locations using standalone applications or recorded media that do not require Internet or intranet connections for the delivery and / or return of the learning materials and activities. The main tasks are usually performed on a PC or laptop.		X	X	X		X
*May be Internet-mediated:* - Audioconference (e.g., telephone only) - Videoconference (e.g., Zoom webinars)	Engaging participants at different locations using synchronous interaction that may, or may not, require Internet or intranet connections for the delivery and / or return of the learning materials and activities.	X	X		X		X
*Internet-mediated:* - e-Mail - Tutorials / online module / interactive modules - Weblinks - Online discussion forum - MOOCs - Social networks	Engaging participants at different locations using the Internet or intranet connections for the delivery and / or return of the learning materials and activities.	X	X	X	X	X	X
*Mobile network mediated:* - Mobile device (e.g., Smartphone, Personal digital assistant (PDA), Tablet)	Engaging participants at different locations using cellular network connections for the delivery and / or return of the learning materials and activities.			X			
*Learning Management System (LMS) facilitated*	Software programmes that facilitate the management (administration, documentation, tracking, reporting, automation) and engagement of participants at different locations for the delivery and / or return of the learning materials and activities			X			X
**2. Enhancing Technology**							
Narrated PowerPoint			X	X			X
Computer Simulation / Virtual Reality	A computer-generated three-dimensional representation of a real or artificial environment that can be interacted with through sensory feedback in a seemingly real or physical way by a person using special electronic equipment, such as a helmet with a screen inside or gloves fitted with sensors.	X					X
Resource access (e.g., library, database, weblink, podcast, video, journal club)	Any single or organised collection of electronic resources that can be accessed by participants at different locations using Internet, intranet, or cellular network connections or recorded media.	X	X	X			X
Scenario-based Learning	Where interactive audio-video scenarios (story lines) are used to support active learning strategies such as problem-based or case-based learning.		X				
^*^“X” indicates one or more of the example modes of delivery or technological approaches were identified within a reference.							

Models such as Knowledge Skills Attitude (KSA) or Knowledge Skills Behaviour (KSB) are used to summarise essential tangible (knowledge and skills) and less tangible (attitude/behaviour) attributes of importance in professional practice [29, 30]. Productive TEET activities will lead to noticeable improvements in KSA/KSB, which can be used as indicators of a successful outcome [24, 25]. Similarly, the satisfaction of learners, which was generally high and which can be measured in various ways, indicates if a TEET activity has met the expectations or needs of the learner, and can also be used as an outcome indicator [25]. All six papers in this study noted the application of KSA / KSB and satisfaction as outcome indicators, and five noted cost or time implications [22, 24–27] or noted barriers to implementation [23, 26, 27]. Impact of CPD on clinical or health outcomes was noted (but described as poorly defined and lacking a standardised model for evaluation), and ‘change in practice’ was also noted as an outcome measure [24, 26] not always with favourable results [22].

### User satisfaction

User satisfaction was reported from three perspectives related to ‘elearning’, ‘videoconferencing’ and ‘readiness’. Satisfaction with elearning was attributed to ease of use, availability of content, content interactivity, reliability, ongoing provision of resources and appropriate support. In relation to videoconferencing, satisfaction was due to the high level of interactivity with facilitators and other participants [22]. However, dissatisfaction was also noted in combined settings, with rural participants feeling isolated and local face-to-face participants resenting having to wait for connection with, and responses from, distant sites [22]. Finally, it was reported that participants' readiness to use the technology also influenced satisfaction [22, 26].

### Changes in health professionals’ performance

Several studies looked at the professionals’ performance by examining changes in knowledge, skills, and behaviour (KSB). TEET led to increased knowledge [22, 25, 26], skills and self-efficacy [22, 23, 25, 26], and leadership skills [25].

### Comparison of different modes of delivery

Blended learning was considered by Rohwer et al. to be more effective than face-to-face learning or no learning intervention [23]. However, Sullivan reported no advantage of blended learning over face-to-face [24]. Pure elearning using different types of learning technologies was considered to be comparable to face-to-face interventions in terms of knowledge and skills, and both were better that than no learning intervention [23, 24].

### Cost

An economic evaluation of CPD was seldom reported [23, 25]. Cost related advantages and disadvantages of CPD were reported. Disadvantages were the cost of technology infrastructure [22, 23, 26], staff and technicians to develop CPD, and Internet connectivity [23, 24]. CPD reduced staff travel costs and time [22–24, 26], and provided cost-effective access to CPD [22, 25]. Videoconferencing for rural practitioners was considered to be cost-effective because of the saving in travel time and costs of being away from work [22]. CPD using elearning was easy to access and use [25]. Sullivan noted the cost of the subscription to online CPD as a barrier, while Berndt et al. identified the benefit of the reduced cost of access to CPD through elearning. The provision of financial incentives to participate in online elearning was also reported [24].

### Other barriers

Challenges to use of elearning were related to poor connectivity, bugs with the systems and limited availability of equipment and systems, time constraints, and the issue of working from home [22, 26]. In addition, some were not ready to use technology and had limited study and computer skills [26].

## Discussion

There was marked heterogeneity of study approach and results in the reviews, making broad interpretation inconclusive [22–27]. Collectively the reviews and their cited papers applied communities of practice or more commonly various learning management systems (LMS such as Blackboard, Moodle) over highly varied lengths of programme time (e.g., 20 min to 6 h, once or over a 3–8 week period) to examine: different groups of healthcare workers (e.g., allied health professionals, nurses, physicians), from different country types (developed versus developing), and from different geographic regions (e.g., North America, Europe, Australasia). They used different modes of delivery (e.g., face to face, blended learning, fully online), addressed various topics (e.g., biostatistics, research ethics, evidence-based healthcare, pharmacology, dementia management, AIDS/HIV, patient safety), leveraged various tools (e.g., multimedia, multiple techniques, multiple exposures, animation, simulation, virtual reality, hyperlinks), and used various means of assessment (e.g., quizzes, pre-test / post-test, surveys) to gauge different comparisons between healthcare worker groups and modes of delivery using a variety of outcome measures (e.g., satisfaction, knowledge gain, change in practice).

Overall the selected reviews showed that technology use is no longer novel [22] and that many different approaches and tools have been applied to provide tertiary and professional education [22–27]. Many learner characteristics (e.g., age, gender, practice setting, experience, speciality, country of training, practice) and external factors (e.g., funding, available infrastructure and infostructure, licensing, accreditation) were noted to influence which tool and approach is best in any given setting. The utility and impact of more recent tools (e.g., smartphones for mobile distance education) did not figure highly in these reviews, but the swift pace of change in technology was noted.

All forms of distance education provided a benefit over no education, with elearning being effective across different learners, learning contexts, clinical topics and learning outcomes. Overall there appears to be little or no distinction to be made between elearning and traditional onsite or face-to-face education, with both being comparable or at least as effective as one another for improving participant knowledge gain, skills and practice decisions, as well as patient outcomes [22]. Despite these generally positive findings there remains uncertainty regarding specific conclusions about which tool and or approach is best for which form of education, for which topic, and for which type of learner. Essentially, there is no ‘one size fits all’ and as a consequence application of blended and / or elearning requires careful planning.

In practical terms, distance education allows a ‘one-to-many’ (synchronous) or ‘none-to-many’ (pre-recorded asynchronous) delivery, allowing efficient access by large numbers of healthcare professionals and others over geographically wide areas. Synchronous versus asynchronous delivery each have benefit. For example, the former allows real-time interaction with instructors and other participants for debate and clarification, while the latter permits ‘any time’ access. Of note was that face-to-face, or at least blended learning, may still be necessary for educational activities that require a change in learners’ values or beliefs in order to permit in-depth debate, or for activities where the practice of new skills is beneficial [22]. Cost and viable connectivity can still be barriers [26].

Less positive aspects were also noted. Whilst distance education avoids the cost and inconvenience of travel and time away from a remote, often single practitioner ‘time poor’ setting, there is still inconvenience and difficulty in making time available when pursuing education ‘at home’ [24]. Dedicated time and space for elearning is not available, potentially requiring use of ‘off work site locations’ [22]. Other issues noted were: bugs in programmes, lag time, need to refresh the Internet connection and limited access to necessary equipment [22]. In addition, participant fatigue was reported when attending protracted activities (e.g., a day-long videoconference activity) indicating shorter periods would be of benefit (e.g., learning sessions of 20–30 min or less), or an ability to save progress and pick up later [22, 26]. The need for consideration of the characteristics of targeted adult learners was also noted, in particular their differing technological expertise and online access capability [24].

Of particular note was the absence of identifying educational needs before implementing programmes [24, 26]. This is related to other generic e-readiness and needs assessment issues beyond elearning, which is of importance in a country like Rwanda that has seized upon the broad use of ICTs as a key national strategy. Studies have shown that the more ‘ready’ a setting is to adopt solutions facilitated by ICT, the more likely implementation is to succeed. Similarly, any ICT solution (including elearning programmes) must be ‘needed’ [24, 26], responding to an evidence-based issue rather than a perceived issue. As a consequence, readiness assessment and needs assessment prior to any elearning implementation will enhance the probability of successful implementation, scaling, and ultimate integration and sustained routine use.

Also of concern is experience in the development of, or conversion to, online formats. Experience of the authors and reports in the literature have shown it is not effective to simply ‘e’ an existing face-to-face module or programme [10]. For example, for asynchronous delivery (absence of a live facilitator) incorporating interactive experiences can be time consuming, and for synchronous delivery (presence of a live facilitator) logistical and audience management difficulties arise when handling a mixed cohort of online and on-campus students.

Based upon the study and author experience, the following recommendations are proffered.

- Identify clear and prioritised health-related CPD educational needs (needs assessment).
- Identify and apply the simplest TEET solutions (mode of delivery, technological approach) to address the identified educational needs.
- Ensure the readiness of the national, facility, and local settings:

- Ensure national infrastructure and infostructure can support the chosen TEET solutions that are feasible and affordable for the given context.
- Ensure the technological literacy of all planned learners.
- Ensure all planned learners have affordable access to programmes, required equipment, and required connectivity.

- Ensure all education interventions are based on sound educational theories or conceptual frameworks, and that appropriate andragogy is used during design and implementation.

- Implement training programmes for educators in the design and provision of TEET for CPD to adult learners.

- Engage national professional societies to design, instigate, and maintain mandatory, incentivised, and regulated CPD programmes.
- Ensure all CPD programmes are evaluated, monitored for ongoing performance, and reviewed and revised periodically (every 3–5 years) to maintain currency and relevance.

## Conclusions

The literature shows that several successful TEET options exist for delivery of CPD to healthcare professionals (pure elearning and blended learning modes, and both Internet-based and non-Internet based technologies). All types of technology approach enhance knowledge, skills and attitudes, and pure elearning is comparable to face-to-face delivery. However, CPD training programmes must be carefully designed and structured. They must have clear goals and objectives and be needs-based, engaging, authentic (linked to real-life practice or realistic scenarios), and interactive. They must also be appropriately designed, evidence-based, utilise multiple delivery methods and multiple exposures, and provide built-in learner self-assessment activities. Finally, they must respect the drivers of independent, self-directed, life-long learners, and apply adult learning principles. The insight and recommendations from this study will be of value to developing countries and Rwanda specifically as CPD for healthcare professionals is further developed in these settings.

## Data Availability

Data are available on request.

## Abbreviations

AIDS: Acquired immunodeficiency syndrome
CBL: Computer-based learning
CBT: Computer-based Training
CD: Compact Discs
CME: Continuing Medical Education
CPD: Continuing professional development
DVD: Digital Versatile Disc or Digital Video Disc
HIV: Human immunodeficiency virus
ICT: Information Communication Technology
KSA: knowledge, skills, and Attitude
KSB: knowledge, skills, and behaviour
LMS: Learning management system
MDGs: Millennium development goals
MOOCs: Massive Open Online Courses
PDA: Personal digital assistant
SDG: Sustainable development goals
TEET: Technology Enabled and Enhanced Teaching
